# Processing, Characterization and Engineering Applications of Fiber-Reinforced Thermoplastic Polymer Composites

**DOI:** 10.3390/polym17111511

**Published:** 2025-05-29

**Authors:** Ankit Gupta

**Affiliations:** Department of Technology, College of Engineering, Computer Science, and Technology, California State University, Los Angeles, CA 90032, USA; agupta26@calstatela.edu

## 1. Introduction

Fiber-reinforced thermoplastic polymer composites (FRTPCs) represent a transformative class of advanced engineering materials that combine the high specific strength and stiffness typical of fiber reinforcements with the inherent advantages of thermoplastic matrices, including rapid processing, recyclability, and thermal weldability. Traditionally, structural composites have been dominated by thermosetting polymers due to their superior load-bearing performance and ease of processing. However, increasing emphasis on environmentally sustainable manufacturing, life-cycle performance, and process flexibility has catalyzed a shift toward thermoplastic-based composite systems.

In FRTPCs, the polymer matrix serves as the load transfer medium and structural binder, while fibers such as glass, carbon, aramid, and natural cellulosic strands provide reinforcement. These systems are particularly attractive for transportation, aerospace, and infrastructure sectors where high mechanical performance must be coupled with weight reduction, damage resistance, and design adaptability. Thermoplastics also enable out-of-autoclave processing techniques such as induction welding, compression molding, and vacuum-assisted forming, making them compatible with high-rate and automated production lines.

Despite these benefits, FRTPCs pose distinct challenges in processing and performance optimization. Key issues include achieving homogeneous fiber distribution, minimizing void formation, ensuring strong fiber–matrix interfacial bonding, and scaling up processing routes while maintaining material integrity. Moreover, accurate characterization of their mechanical behavior, thermal stability, and wear resistance under service-relevant conditions is essential for performance validation and certification.

Recent developments in filler functionalization and hybrid reinforcement strategies have further expanded the functionality of FRTPCs. The incorporation of nanoscale reinforcements—such as graphene derivatives, carbon nanotubes, and metal oxides—has yielded composites with enhanced conductivity, self-sensing capabilities, and tunable mechanical responses. These advances enable the design of multifunctional materials for emerging applications in flexible electronics, energy harvesting, and structural health monitoring.

This Special Issue compiles contemporary research focused on the synthesis, structural engineering, and practical utilization of FRTPCs. It aims to present both experimental and theoretical contributions that address processing complexities, performance evaluation, and integration strategies for next-generation composite systems.

## 2. Overview of the Published Articles

This Special Issue comprises 13 rigorously peer-reviewed articles that collectively reflect the diversity and technical depth of ongoing research in the field of fiber-reinforced thermoplastic polymer composites (FRTPCs). These contributions span a broad range of topics, including material synthesis, advanced manufacturing techniques, structural performance evaluation, and functional integration for application-specific requirements.

A subset of the papers is centered on the development and processing of bio-based and biodegradable polymer systems aimed at addressing sustainability while maintaining or enhancing the mechanical performance of composites. Several studies focus on the incorporation of reinforcing agents such as nanofillers, short and continuous fibers, and hybrid additives to improve the stiffness, strength, and thermal resistance of thermoplastic matrices. Other studies emphasize application-driven innovations in packaging, infrastructure, transportation, and biomedical fields, merging foundational material science with scalable implementation.

Xu et al. presented a detailed thermal and mechanical analysis of glass-fiber-reinforced polypropylene rebars, emphasizing their thermal softening behavior and processing flexibility for field applications. Rocha et al. demonstrated the fabrication of a composite spar via oven vacuum bagging, offering an economically viable and environmentally friendly approach to large-scale thermoplastic component manufacturing. Li et al. analyzed the dynamic behavior of precast bridge columns enhanced with basalt–FRP under seismic loads, providing a performance-based validation of thermoplastic composites in civil infrastructure. Takayama et al. explored the role of carbon nanofiber dispersion in aramid–polypropylene matrices, linking microstructural uniformity to enhanced load-bearing capacity. Choi et al. investigated secondary bonding in aircraft-grade composites through the press conduction welding of PEKK-based laminates, providing a practical solution for assembling complex aerospace structures. Kaya introduced a novel natural fiber sourced from Platanus orientalis L. stems, characterizing its mechanical and chemical properties and evaluating its suitability for sustainable biocomposites. Li et al. developed PEEK fiber-reinforced friction materials through wet granulation, reporting significant improvements in wear and heat resistance, critical for automotive applications. Nishi et al. applied electron beam irradiation to modify polycarbonate/carbon fiber composites, resulting in improved flexural performance. Yu et al. evaluated the mechanical behavior of multilayered self-reinforced polyethylene composites, revealing structure–property correlations driven by layer configuration. Agbozouhoue et al. examined fungal degradation effects on ZnO-treated polyethylene/yellow birch fiber composites, assessing the long-term stability of bio-hybrid systems. Ciardiello et al. optimized the performance of glass-fiber-reinforced composites using recyclable thermoplastic infusion resins, supporting circular material economies. Xu et al. implemented electromagnetic induction heating for CFRP tube fabrication, offering insights into energy-efficient thermal processing. Lastly, Albuja-Sánchez et al. provided a comprehensive review of fiber-reinforced polymer applications in civil infrastructure, with a focus on material longevity, corrosion resistance, and fast-track deployment in rehabilitation projects. The seismic performance of precast bridge piers can be strengthened using basalt–FRP, offering solutions for resilient infrastructure.

## 3. Summary and Future Outlook

The advancements presented in this Special Issue underscore the transformative potential of high-performance thermoplastic composites in addressing complex engineering challenges across diverse industrial domains. The featured studies collectively highlight a trajectory toward sustainable, multifunctional, and process-efficient composite systems, capable of meeting both structural and functional performance benchmarks. One of the dominant themes observed across the contributions is the refinement of advanced manufacturing techniques. Approaches such as press conduction welding, electromagnetic induction heating, and oven vacuum bagging are being adapted to enhance the scalability, precision, and quality of thermoplastic composite fabrication. These techniques are critical not only for improving throughput in industrial settings but also for enabling the production of large and geometrically complex components.

Several works place a strong emphasis on the development of environmentally friendly material systems, particularly through the incorporation of bio-based reinforcements and recyclable thermoplastic matrices. These efforts reflect a growing commitment to life-cycle sustainability and the integration of green chemistry principles into composite design. The collection also showcases significant strides in addressing microstructural and interfacial challenges that influence composite performance. Innovations in fiber–matrix compatibility, nanoscale filler dispersion, and hybrid reinforcement strategies have yielded composites with enhanced mechanical robustness, thermal stability, and wear resistance. Notably, smart functionalities—such as thermally responsive behavior, energy dissipation capabilities, and self-adaptive mechanical properties—are being embedded into structural composites, positioning them for future use in intelligent material systems.

Looking forward, key research directions include the standardization and reliability assessment of emerging processing methods, exploration of long-term material performance under coupled environmental and mechanical loads, and the development of multifunctional composites with tailored property profiles. As thermoplastic composites continue to intersect with digital design, environmental policy, and advanced manufacturing, they are poised to redefine material innovation for next-generation engineering applications.

